# Practical Medicine and Therapeutics

**Published:** 1837-04

**Authors:** 


### PRACTICAL MEDICINE AND THERAPEUTICS.

### *On Dropsy.* By Dr. Mateer, of Belfast.

There is a form of dropsy which occurs in debilitated persons without any well-marked symptoms of organic disease: the older writers attributed it to laxity of the exhalents, but Dr. Mateer's theory is, that it depends on diminished action of the kidneys, or asthenic anæmia of these organs. It appears to be very prevalent among the Irish poor. Out of 1844 patients prescribed for at the Belfast Dispensary in 1833, there were 184 affected either with anasarca or ascites, in whom no organic disease could be detected; the urine was incoagulable. In these cases, (says Dr. M.) there were often symptoms simulating organic derangement; such as bitterness of the mouth, pain of the right side, slight cough, flatulence, costiveness, palpitations, and flutterings at the epigastrium; but these he considered as merely symptomatic of general debility, and they yielded readily to tonics. The causes of this form of dropsy, are, innutritious food, cold, and fatigue, and sometimes blood-letting carried to a great extent.

Dr. Mateer's treatment consisted in general and local stimulants: of the latter, the most beneficial were saline medicines, and of these the best are carbonate of ammonia and supertartrate of potash. In using saline medicines, it is not uncommon for the urine to become coagulable; and, as Dr. Burrows, jun., has remarked, this fact has been overlooked, from these medicines when excreted having the power of dissolving coagulated albumen, and thus masking it, if heat be the only test employed. If salines disagree with the stomach, tincture of cantharides, or oil of turpentine, may be substituted for them.

Dr. Mateer's reasons for believing this kind of dropsy to depend on anæmia of the kidneys are founded on reasoning alone. They are, 1. From the treatment: stimulant diuretics carry off the effusion, and, in doing so, they cause a temporary albuminous state of the urine by irritating the kidneys. 2. As the office of the kidneys is to excrete superfluous fluid, their imperfect action would cause a more fluid state of the blood than natural; and hence the secretion of serum into the cellular tissue. 3. Andral and other pathologists have admitted that such a state of the kidney sometimes exists.

*Edinburgh Medical and Surgical Journal, January,* 1837.

---

### *A Letter on Typhus Fever.* By Robert Perry, M.D., of Glasgow.

This communication refers principally to the statements and views of Dr. Lombard, noticed in our last Number, p. 260. We must content ourselves with one extract from it, which cannot fail to interest the reader, even although he may not assent to all the author's views.

"I have, for some years, entertained the opinion, founded upon an extensive series of observations, that contagious typhus is an *exanthematous disease,* and is subject to all the laws of the other exanthemata;* that, as a general rule, it is only taken once in a life-time, and that a second attack of typhus does not occur more frequently than a second attack of small-pox, and, judging from my own experience, less frequently than a second attack of measles, or scarlet fever.

"It is a very generally received opinion, that typhus fever may originate spontaneously, and become contagious from filth, improper diet, impure air, from the confinement and crowding together of human beings, or from some peculiar constitution of the atmosphere, or emanation from the bowels of the earth. Where are the well attested facts to be found, which can warrant any reliance on such

* "In proof of this, I have often, in cases shewing symptoms of typhus, and when I learned that they had been exposed to contagion, and had not been secured against by a previous attack, predicted with unfailing certainty, the appearance of the typhous eruption on the fifth, or at furthest the sixth, day from the commencement of the attack."

fancies, fancies so contrary to sound philosophy? To suppose that any of these causes could generate a specific poison, and this always of the same kind and character, capable of propagating itself by contagion, requires stronger faith than I am possessed of; nothing but the most positive evidence could make me for a moment entertain such an opinion. It is, undoubtedly, a matter of great practical importance, to ascertain exactly the period of the disease at which typhus fever becomes contagious. From numerous observations and experiments I am satisfied, that it is not contagious before the ninth day, perhaps not till a later period of the disease. Among many circumstances which establish this opinion, I may mention one experiment which I made upon a pretty extensive scale. The fever wards of the Glasgow Royal Infirmary are each capable of containing twenty patients. The beds are arranged in two opposite rows, and are pretty near each other. While the patients are in the acute wards, they are not allowed the use of their clothes, though they may be able to sit up; they are, therefore, almost constantly confined to bed, excepting when rising to stool; and there is about one close-stool to every three patients. Into the fever house are admitted, cases of measles, scarlet fever, and small-pox; and patients are very frequently sent in, labouring under bronchitis, pneumonia, erysipelas, and other local inflammatory affections. I found by experience, that when the latter class of patients were sent to the convalescent ward, where they necessarily mixed with the others, almost all those who had not a previous attack of typhus fever were either seized with it before leaving the house, or returned soon after their dismissal, labouring under it; the period intervening between the time of their being sent to the convalescent ward, and the attack, never being less than eight days. Although means were taken to keep those recovering from small-pox, scarlatina, &c., in a separate room from those convalescent from typhus, the rooms being adjoining, the non-intercourse was incomplete, and the result was, that these diseases occasionally spread among the typhus convalescents, and the convalescents from small-pox and scarlatina caught typhus. In consequence of these observations, I adopted the practice of not sending, as formerly, to the convalescent wards, those patients affected with inflammatory diseases, unless I ascertained that they were secured against the disease by having had a previous attack of typhus; but kept them in the acute fever wards till they were so far recovered as to go to their own houses, and the result was, (and the practice was continued for several months,) that not one of those detained in the acute wards caught the disease while there, or returned with it afterwards. From the above, and other observations, I have adopted the opinion, that typhus, like measles, small-pox, &c., is chiefly spread during the period of convalescence." *Dublin Journal*, January, 1837.

---

*Case of Ulceration of the Brain.* By A. J. HANNAY, Professor of Physic, Anderson's University, Glasgow.

THIS case is more remarkable for the rapidity of its course than for the appearances found on dissection, although these (unequivocally those of ulceration of the brain,) are far from common. A boy, æt. nine, who although delicate had been sufficiently well to attend school, until three days before the day (22d February) he was seen by Dr. Hannay labouring under all the usual symptoms of inflammation of the brain, followed by effusion. He had extreme pain of the head, with fever and with quick pulse (116), at first. On the 23d the pulse fell to sixty-eight; on the 26th to twenty-eight; on the 27th it became again quick. He died on the 28th. Dissection disclosed the usual appearances of arachnitis, and an ulcer in the cerebral substance. No doubt the latter had been of some standing, and was the exciting cause of the fatal attack of arachnitis. The ulcer is thus described by Dr. Hannay:

"On the surface of the left anterior lobe, on its outer and under side, there was a cavity or excavation of the convolutions, partly smooth, partly granulated, of a pale primrose hue on some parts, and of the cineritious colour of the brain in others. The granulations were small, whitish, shining bodies, and gave to the surface of the cavity the appearance of being sprinkled over with oatmeal in a moist state. Over part of the surface there was a cobweb-like expansion of small vessels: this was principally on the scabrous or granulated part. The cavity was of an irregular oval shape, with an area greater than that of a shilling; its edges were more than an eighth of an inch in depth, and fringed with the membranes which had been destroyed by the ulceration; its surface was as soft as thin paste or starch, but not diffluent." *Dublin Journal, January,* 1837.

---

*Observations on Cerebral and Spinal Apoplexy, Paralysis, and Convulsions of New-Born Infants.* By EVORY KENNEDY, M.D., Master of the Dublin Lying-in Hospital.

THE celebrated Lying-in hospital in the Irish metropolis, from the records of which practical medicine has already derived so great advantages, is not likely to be barren of important results in the hands of the present master, if we may judge from the valuable paper before us. This commences with some very judicious observations on the obscurity of diagnosis in infantile diseases, particularly in affections of the cerebro-spinal system; the author justly concluding that "the most elaborate and accurate inquiries into cerebro-spinal pathology at the present day have arrived at little more than the threshold of this subject, as far as establishing a fixed relation between diagnosis and disease." Convinced that improvement in this path can only be attained "by the candid relation of what has been absolutely observed," without "embarrassing the mind of the enquirer by crude of hypothesis," Dr. Kennedy, in the present memoir, "does little more than afford a detail of actual observations made upon cases of these obscure diseases falling under his notice." As these cases, nineteen in number, are not susceptible of abridgment, we can only here indicate their general character, recommending the reader to the perusal of them in the original. We must, however, extract the explanation given by the author of the cause of the frequency of these affections in infants:

"The causes of the predominance of apoplexy in the new-born infant may be ascribed, first, to the so suddenly altered circulation, by which the influence of the maternal circulating system upon the fœtus is done away with, whilst the nice adjustment necessary in the permanent establishment of respiration, an independent circulation, and the right performance of the cerebro-spinal functions, but more especially of their mutual relations and dependencies, is still scarcely completed. The balance that time afterwards fixes so accurately between these important vital organs, as essential to the very existence of the individual, is still unsettled; and, from this circumstance, very slight causes will produce excessive derangement. The lungs, also, at this period are expanded but in part, and a very important change is effected in the circulation of the blood in the heart and great vessels by the closure of the foramen ovale, and the current passing from the ductus arteriosus. From these several causes, then, the blood is frequently thrown in excess upon the brain and lungs, or retarded in its transmission through them. The coats of the arteries are thin and yielding, and, from deranged vital energy in the still imperfectly established functions of the ganglionic system supplying them, the due transmission of the blood may be further interfered with. These considerations, with a recollection of the structure of the brain in the fœtus, which is so soft and vascular; of the compression in the dependent position to which it is liable in birth; and the difficulty and delay often attendant on the first establishment of respiration, will tend rather to excite our astonishment that cerebral affections in the infant should not be more numerous than they are."

I. *Cerebral Apoplexy in New-born Infants.*

Case 1st. Primary or simple apoplexy. 2d. Apoplexy with tonic spasm. 3d. Secondary apoplexy. 4th. A. from obstructed respiration. 5th. A. from mechanical malposition of the viscera.

II. *Spinal Apoplexy of New-born Infants.*

Case 6th. Trismus. 7th. Congestive A. of the spine. 8th. Ditto. 9th. Effusion into the spinal canal.

III. *Paralysis in New-born Infants.*

Case 10th. Paralysis of the seventh pair of nerves. 11th. P. of the portio dura of right side, and third nerve of left. 12th. P. of portio dura, and spinal nerves of right side. 13th. Hemiplegia of left side, &c.

IV. *Convulsions of New-born Infants.*

Case 14th. Convulsion of muscles of deglutition, with catalepsy. 15th. Convulsions from deranged bowels. 16th. Convulsions preceding miliary eruption. 17th. Do. from scrofulous eruption. 18th. Do. with serous effusion into cranium and spinal cord. 19th. Convulsions followed by apoplexy.

*Dublin Journal, January,* 1837.

---

*Researches on the Symptoms and Diagnosis of Aneurismal and other Tumours in the Cavity of the Thorax.* By GEORGE GREENE, M.D., Lecturer on the Practice of Medicine at the Richmond Hospital School, Dublin.

THIS paper, and another on the same subject in a former Number of the Dublin Journal, forms a valuable addition to our knowledge of thoracic pathology and diagnosis. The present memoir contains a detailed account of three cases, with the appearances on dissection, and many judicious remarks, observations, and inferences; all of which are well deserving perusal. We can only find room for the titles of the cases, and some general conclusions which they and those formerly published authorize the author to deduce.

CASE I. Intense dyspnœa and bronchitis: an enlarged bronchial gland, containing ossific and cretaceous matter, communicating with the right bronchus.

CASE II. Double bronchitis with intense dyspnœa; emphysema of the cellular tissue of both sides of the neck and left side of the trunk; an abscess in an enlarged bronchial gland, communicating with the left bronchial tube.

CASE III. Aneurism of the transverse portion of the thoracic aorta; sudden death from extravasation of the contents into the left pleural cavity.

The result of these cases, and of others formerly published by the author, appears to him to warrant the following conclusions with respect to tumours developed in the thoracic cavity.

"1st. That no single physical sign, taken by itself, can characterize the nature of any particular tumour.

"2d. That in many cases the presence of an abnormal pulsation in the chest, when detected by the stethoscope, affords the best indication of the existence of an aneurism, and may frequently serve to distinguish it from other tumours attended with nearly similar physical signs.

"3d. That the same rational symptoms, as they have been termed, may be produced by any species of tumour within the cavity.

"4th. That the morbid contents of enlarged scrofulous glands are sometimes evacuated through the air passages in adults, as well as in the earlier periods of life; and that during the process, death may occur from excessive dyspnœa and bronchitis, or from general emphysema.

"The analysis of the physical signs and general symptoms, observed in the above case of aneurism, and of three others which I formerly published, is as follows. In all these four cases the disease was situated in some portion of the arch of the aorta.

"*First of the Physical Signs.* A single or double impulse in situations more or less distant from the heart was discovered by auscultation in the four cases.

"Two sounds, more or less distinct, accompanied this impulse. A bruit de soufflet was heard in two, and a bruit de rape in one case.

"A feebleness of the respiratory murmur was observed in every case in one or other of the lungs; while over the same lung a clear sound was obtained on percussion.

"Bronchial rales of greater or less intensity were heard throughout both lungs in each case.

"An impediment to the descent of the morsel existed in three cases, and was referred by the patients to situations nearly corresponding to the sterno-clavicular articulation on the right side.

"*Second of the General Symptoms.* Pains of a lancinating or burning character around the parietes, or through the centre of the chest, were much complained of in all the cases; they were sometimes remittent, and relieved by pressure.

"Dyspnœa or orthopnœa existed in every case. In one only was the dyspnœa inconsiderable.

"A cough, usually of a loud, ringing, or croupy character, occurring in paroxysms, existed in each case. In three the above characters were very remarkable, in one not so much so.

"Turgescence of the jugular veins was observed in all the cases. And in two, tenderness of the spine on pressure.

"The symptoms which sometimes attend aneurism of the arch, but which were not observed in any of these cases, are as follows:—Numbness, cramps, tingling pains, œdema, or loss or difference of temperature in the upper extremities. Neither was there any difference in the pulse at either wrist."

*Dublin Journal, January,* 1837.

---

*Poisonous Effects of Black Oxide of Manganese.* By John Cowper, M.D., Professor of Materia Medica, Glasgow.

Professor Cowper briefly notices several cases of disease which took place among men employed in grinding the black oxide of manganese, to be employed in the manufacture of bleaching powder. These cases seem to show that manganese is an active poison, and that its effects, when slowly introduced, resemble those of mercury and lead. Like these metals, it paralyzes the motor nerves; but it differs from mercury, by first affecting the lower extremities, and by not occasioning tremors of the affected part; and from lead it differs, by not affecting the intestinal canal: thus it gives rise neither to the *tremblement metallique* of the former, nor the *colica pictonum* of the latter. The disease produced by manganese, like that by the other metals, is very permanent and intractable.

*British Annals of Medicine, January* 13, 1837.

---

*Third Report of Medical Cases treated at St. George's Hospital,* [*during the year* 1836.] By R. Macleod, M.D.

This Report is very creditable to the industry and intelligence of the author; and its publication is the more praiseworthy from the fact stated by Dr. Macleod, that he has "not yet had the satisfaction of seeing reports of the results of their practice from any other of the medical officers of the metropolitan hospitals." The Report contains the histories of some interesting cases and also important practical remarks; but we can find room only for the chief statistical details.

"On the 1st of January, 1836, the patients in the hospital, under my care,

| | | |
|---|---|---|
| amounted to | | 23 |
| There have since been admitted | | 254 |
| Total to be accounted for | | 277 |
| Of the above cases there have been cured | 168 | |
| Relieved | 35 | |
| Complaints stationary | 10 | |
| Transferred to surgeon | 4 | |
| Dead | 38 | |
| Remain under treatment | 22 | |
| Total | 277 | |

The following table exhibits the ages of the 277 patients treated in 1836, and the number of deaths in each decimal period.

| | | | Deaths. |
|---|---|---|---|
| | Under 10 | 5 | 0 |
| Between | 10 and 20 | 42 | 3 |
| .. | 20 .. 30 | 80 | 5 |
| .. | 30 .. 40 | 67 | 8 |
| .. | 40 .. 50 | 39 | 6 |
| .. | 50 .. 60 | 29 | 8 |
| .. | 60 .. 70 | 12 | 7 |
| .. | 70 .. 80 | 2 | 1 |
| .. | 80 .. 90 | 1 | 0 |
| Total | | 277 | 38 |

"The increase of mortality with the advance of years is rendered very striking by the above table. Of the five deaths between the ages of 20 and 30, two were from poisoning; and if we deduct these, so as to retain only cases of disease, we have 78 patients yielding but three deaths; whereas, between 60 and 70, of twelve patients, not fewer than seven died—the diseases being, organic affections of the heart, with dropsy, 2; organic affections of the liver, with dropsy, 2; phthisis pulmonalis, 2; bronchitis, 1.

*Cerebral Diseases*—14.

Apoplexy, with hemiplegia, 3.—Cured, 2; dead, 1.

Hemiplegia, without apoplexy on admission, 4.—Relieved, 1; stationary, 2; dead, 1.

Inflammatory affections of the encephalon, 4.—Cured, 3; dead, 1.

Epilepsy, 2.—Cured, 2.

Mania e potu, 1.—Cured, 1.

*Spinal Diseases*—6.

Paraplegia, 3.—Cured, 1; relieved, 1; dead, 1.

Paralysis of fore arms, 3.—Relieved, 2; stationary, 1.

*Various Nervous Diseases*—30.

Hysteria, 17.—Cured, 10; relieved, 5; remaining, 2.

Chorea, 7.—Cured, 6; remaining, 1.

Paralysis agitans, 1.—Much relieved, 1.

Neuralgia, 2.—Cured, 1; relieved, 1.

Hypochondriasis, 3.—Relieved, 2; stationary, 1.

*Diseases of Respiratory Organs*—35.

Phthisis pulmonalis, 14.—Relieved 8; dead, 6.

Bronchitis, 8.—Cured, 5; dead, 2; under treatment, 1.

Laryngitis, 1.—Cured, 1.

Pulmonitis, 4.—Cured, 3; dead, 1.

Pleuritis, 2.—Cured, 2.

Hæmoptysis, 3.—Cured, 3.

Sloughing abscess of lung, 1.—Dead, 1.

Empyema, 1.—Under treatment, 1.

Hooping cough, 1.—Cured, 1.

*Diseases of the Heart and its Appendages*—24.

Acute pericarditis, 6.—Cured, 4; relieved, 1; dead, 1.

Various organic diseases of the heart, with dropsy, 16.—Cured (as regards the dropsy), 6; dead, 6; stationary, 1; under treatment, 3.

Organic diseases unaccompanied by dropsy, 2.—Relieved, 1; under treatment, 1.

*Diseases of the Fauces*—2.

Cynanche tonsillaris, 2; cured, 2.

*Diseases of the Abdominal Viscera*—51.

Indigestion, without evidence of organic disease, 7.—Cured, 5; relieved, 2.

Ditto, dependent on organic disease, 3.—Stationary, 2; dead, 1.

Hæmatemesis (vicarious), 3.—Cured, 3.
Ditto, from diseased liver, 1.—Dead, 1.
Vomiting and purging (English cholera), 2.—Cured, 2.
Dysentery, 6.—Cured, 3; relieved, 1; under treatment, 1; dead, 1.
Painter's colic, 2.—Cured, 2.
Enteritis, 1.—Cured, 1.
Icterus, 1.—Cured, 1.
Diseases of liver, with more or less of hepatic inflammation, 12.—Cured, 7; relieved, 2; under treatment, 3.
Chronic diseases of liver, with ascites, 13.—Cured, 2; under treatment, 3; stationary, 1; dead, 7.

*Diseases of the Kidneys*—6.

Nephralgia, 2.—Cured, 1; under treatment, 1.
Organic diseases, with dropsy, 4.—Cured (as regards the dropsy), 2; under treatment, 1; dead, 1.

*Diseases of the Uterine System*—11.

Hysteritis, 1.—Cured, 1.
Flooding after abortion, 1.—Cured, 1.
Menorrhagia, 4.—Cured, 3; relieved, 1.
Amenorrhœa, 2.—Cured, 1; under treatment, 1.
Ovarian dropsy, 2.—Stationary, 2.
Malignant tumour in pelvis, 1.—Dead, 1.

*Diseases of the Articular and Fibrous Systems*—56.

Rheumatism (acute), 32.—Cured, 30; under treatment, 2.
Ditto (chronic), 20.—Cured 14; relieved, 6.
Gout, 1.—Cured, 1.
Periostitis, 3.—Cured, 3.

*Exanthemata, and Acute Diseases of the Skin*—9.

Erysipelas of face and head, 4.—Cured 2; dead, 2.
Scarlatina, 1.—Cured, 1.
Rubeola, 1.—Cured, 1.
Pompholix, 1.—Cured, 1.
Urticaria, 1.—Cured, 1.
Purpura hemorrhagica, with apoplexy, 1.—Dead, 1.

*Fevers*—23.

Intermittent, 5.—Quotidian 2; tertian, 3; cured, 5.
Continued, 18.—Cured, 16; convalescent, 2.

*Dropsy, not apparently connected with Organic Disease*—3.

Cured, 2; dead, 1.

*Cases of Poisoning*—3.

With arsenic, 2.—Dead, 2.
With oxalic acid, 1.—Cured, 1.

*Med. Gazette, February* 4, 1837.

---

*On Pectoriloquy.* By EDWIN HARRISON, M.D.

IN this short communication, Dr. H. points out the importance of defining more carefully than has been done by Laennec and many of his followers, the exact import of the sign Pectoriloquy. He states justly that, in cases of decided tuberculous excavation of the lungs, the voice is *not* always transmitted up the tube of the stethoscope; while, in other cases, where there is no cavity, (as in consolidation of the lung around a large bronchus,) there sometimes is transmission of the voice. Dr. H. is of opinion that the most valuable auscultatory sign of a cavern (as far as regards the voice,) is "the resonance of the voice in a circumscribed space;" "a sensation that the space in which the voice plays is circumscribed; such space not existing naturally in the part of the lung from which the sensation is communicated." We have always been of opinion that the value of pectoriloquy, as a sign of phthisis, has been overrated; and we think the observation of Mr. Harrison calculated to render it at once more precise and valuable.

*Med. Gazette, December* 24, 1836.

---

*Remarks on the Nature and Treatment of Rheumatism.*
By J. HOPE, M.D. F.R.S.

THE only part of this interesting paper which we shall notice is the *Treatment of the Acute Rheumatism.* Dr. H. reviews the principal plans which have been employed in this disease, viz. 1, the bleeding and purging, or pure antiphlogistic plan; 2, the forced sweating plan; 3, the stimulant plan, (bark, guaiacum, &c.); 4, the colchicum plan; 5, the calomel and opium plan; and gives a decided preference to the last, not, however, as originally proposed by Dr. Hamilton, but as modified (Dr. Hope says,) by Dr. Chambers, of St. George's Hospital. Dr. Hope says he has employed this plan during the last six years in about two hundred cases, and thus details it and its advantages:

"1. *Acute Rheumatism.* After a full venesection, or even two, in the robust, but without bleeding in the feeble and delicate, I give every night gr. vij. to x. of calomel with jss. to ij. of opium, according to the age, and the severity of the case; and every morning a full haust. sennæ, to act four or five times at least. In addition I generally give the following draught, thrice a day, as it has appeared to me to expedite the cure—partly, perhaps, by the additional opiate, and partly by the sedative effect of the colchicum.—R. Vini colch. m. xv. ad xx.; Pulv. Doveri, gr. v.; M. salin. ℥x.; Syrupi, ʒj. M.

"When the pain and swelling are greatly abated, if not almost gone, (which often happens within two days, and almost always within four,) I omit the calomel; or if the gums become in the slightest degree tender, I omit it even earlier. The opium I continue to the extent of gr. j. or jss. at bed-time; and in severe cases I add a grain at noon; for without an anodyne the pains are apt to recur. I also continue the colchicum draught and the haust. sennæ, as before. No local treatment is necessary beyond warm or cold application, according as the patient finds them agreeable. If the patient is not well in a week I consider it a case of exception; and the exceptions are generally in those who are subject to rheumatism, and who therefore usually have it in a more obstinate chronic form.

"The advantages of this plan are, 1, that a patient is generally sound, well, and fit for work in a week or ten days after the pains have ceased; 2, that the gums are rarely affected, especially if you previously ascertain that the patient has not a morbid susceptibility of mercury; 3, that it is rare to see inflammation of the heart if the treatment is early begun (I think that one case in a dozen would be the maximum in my practice); 4, if the slightest symptom of endo or peri-carditis *does* supervene, a few extra doses of calomel and opium, as gr. v. c. op. gr. j. every four or six hours, will generally affect the constitution in twenty or thirty hours, which, with two or three cuppings or leechings on the region of the heart, almost always places the patient in a state of safety. I have never lost a patient by rheumatic pericarditis since I employed this plan; and I have been told by other hospital practitioners that they have been equally successful by the use of calomel and opium.

"2. *Active Chronic Rheumatism.* Here calomel and opium may be given in smaller doses, as calomel, gr. v. and opium, gr. j. every night; but they require to be continued for a longer time, as five or six nights. Take care, however, to stop short of ptyalism, especially in the scrofulous. Local treatment, too, is more beneficial than in the acute form: viz. bleed locally rather than generally, and ultimately employ blisters, liniments, &c.

"*Trials of Modifications.* I have tested this plan by successively omitting the venesection, the purging, the calomel, and the opium: and with each omission I have found the recovery less expeditious and certain. I cannot doubt that the opium contributes importantly to the cure, perhaps by allaying the pain, and thus diminishing the irritative fever dependent on it; or, possibly, by modifying the vital state of the blood: but this is yet hypothetical. I have, however, assured myself of the fact, that opiates and purging alone will cure many cases of acute rheumatism remarkably well." *Med. Gazette, February* 25, 1837.

---

*Case of Perforation of the Stomach.* By J. C. Parkin, Surgeon, Bridgewater.

This was a case of perforation, occurring in a young woman who had laboured under symptoms of diseased stomach for a year and a half, marked chiefly by pain after eating, particularly if the food was not easily digestible, and the symptom commonly termed *pyrosis*. The most peculiar feature in the case was the very slight effect produced on the system by the local disease of the stomach; the general appearance of the patient, at the time of the accident, being that of a person in perfect health. This is, however, not very uncommon in simple ulceration of the stomach, as was the case here. She survived the rupture about twenty-four hours. The abdominal viscera were found coated with lymph recently effused. The perforation was about half an inch in diameter, about two-thirds distant from the pylorus, and one-third from the œsophagus. The pyloric half of the stomach, besides the large ulcer in which the perforation had occurred, presented numerous smaller ulcerated spots, "as if portions of mucous membrane had been pecked out by a bird." *Lancet, Dec.* 3, 1836.

